# Development of a new gene expression vector for *Thermus thermophilus* using a silica-inducible promoter

**DOI:** 10.1186/s12934-020-01385-2

**Published:** 2020-06-08

**Authors:** Yasuhiro Fujino, Shuichiro Goda, Yuri Suematsu, Katsumi Doi

**Affiliations:** 1 Institute of Genetic Resources, Faculty of Agriculture, Kyushu University, 744 Motooka, Nishi-ku, Fukuoka, 819-0395 Japan; 2 Graduate School of Science and Engineering, Soka University, 1-236, Tangi-cho, Hachioji-shi, Tokyo, 192-8577 Japan

**Keywords:** Silica, Gene expression, Inducible, *Thermus thermophilus*

## Abstract

**Background:**

Thermostable enzymes are commonly produced in mesophilic hosts for research and bioengineering purposes. However, these hosts do not overexpress the active forms of some biologically functional thermoenzymes. Therefore, an efficient thermophilic expression system is needed. *Thermus thermophilus* contains an easily manipulable genome and is therefore among the best candidate microbes for a “hot” expression system. We previously identified a strong and inducible promoter that was active in *T. thermophilus* under supersaturated silica conditions. Here, we report a new heterologous gene expression system based on a silica-inducible promoter in *T. thermophilus*.

**Results:**

A *Thermus* sp. A4 gene encoding thermostable β-galactosidase was cloned as a reporter gene into the expression vector pSix1, which contains a selection marker that confers thermostable resistance to hygromycin and a 600 bp DNA region containing a putative silica-inducible promoter. β-galactosidase activity was 11-fold higher in the presence than in the absence of 10 mM silicic acid. SDS-PAGE revealed a prominent band corresponding to 73 kDa of β-galactosidase, and this enzyme was expressed as an active and soluble protein (yield: 27 mg/L) in *Thermus* but as an inclusion body in *Escherichia coli*. Truncation of the putative silica-inducible promoter region in *Thermus* expression vector improved the yield of the target protein, possibly by avoiding plasmid instability due to homologous recombination. Finally, we developed an expression vector containing the pSix1 backbone and a 100 bp DNA region corresponding to the silica-inducible promoter. We used this vector to successfully express the active form of glutamate dehydrogenase from *Pyrobaculum islandicum* (*Pis*GDH) without additional treatment (yield: 9.5 mg/L), whereas the expression of active *Pis*GDH in *E. coli* required heat treatment.

**Conclusion:**

We successfully expressed the thermostable β-galactosidase and *Pis*GDH in *T. thermophilus* as active and soluble forms and achieved with our system the highest known protein expression levels in this species. These thermoenzymes were expressed in active and soluble forms. Our results validate the use of our silica-inducible expression system as a novel strategy for the intracellular overexpression of thermostable proteins.

## Background

Enzymes from thermophiles are highly stable and have attracted significant interest in both basic research and bioengineering applications [1–3]. Currently, thermostable enzymes used in these context are produced commonly in mesophilic hosts [4], especially *Escherichia coli*. These surrogate hosts can successfully express soluble forms of the target proteins. Although the ability to remove mesophilic host proteins by simple heat denaturation during the production process is advantageous, currently only a few biologically functional thermoenzymes are overexpressed in their active forms in mesophilic hosts. Moreover, these target proteins often aggregate to form “inclusion bodies” that are susceptible to misfolding when expressed at relatively lower temperatures than those experienced in their native hosts [5]. Consequently, the use of thermophilic hosts for enzyme production requires the development of genetic tools that can overcome these limitations.

*Thermus* is among the most ubiquitous genera of thermophilic bacteria. *Thermus* species are characterized by high growth rates and cell yields in culture and a high level of natural competency, and thus exhibit strong potential for production in laboratory settings [1]. Although several studies have demonstrated the potential use of these organisms as cell factories [5–9], only moderate levels of protein overexpression were achieved except in studies that used the expression vector pMKE1, which utilizes the respiratory nitrate reductase operon promoter (*Pnar*). Moreover, despite the achievement of up to 200-fold overexpression levels with this *Pnar*-based expression system in facultative anaerobic derivatives of *T. thermophilus* HB27 (HB27::*nar*), the resulting proteins were almost undetectable by Coomassie brilliant blue staining. To date, successful expression at practical levels has only been achieved using pMKE2, a variation of pMKE1 that was created by modifying the sequences between *Pnar* and the start codon [7]. Recently, Aulitto and co-workers achieved a high level of homologous α-galactosidase production (5 mg/L) in *T. thermophilus* HB27 under the control of pMKE2 [10]. However, no report has described a sufficient level of heterologous expression in *Thermus* cells.

Despite the noted challenges, overexpression in *T. thermophilus* remains a potentially useful tool for the overexpression of thermostable enzymes or the selection of thermostable mutants from mesophilic proteins via directed evolution. We considered that these challenges might be resolved by a strong promoter that would enable more effective expression. We previously reported the expression of a specific silica-induced protein (Sip) in *T. thermophilus* exposed to supersaturated silica [11]. We further identified this Sip as a Fe^3+^-binding ABC transporter encoded by a gene for which the transcription was regulated strictly by a ferric uptake regulator (Fur) via a previously reported mechanism [12]. Importantly, the *sip* promoter might be sufficiently strong to mediate heterologous expression, as indicated by the greater than tenfold increase in Sip expression under induced conditions relative to un-induced conditions and the prominent protein band of 35 kDa detected by Coomassie brilliant blue staining.

The production of active forms of thermoenzymes is important for both enzymological studies and industrial applications. A good example is glutamate dehydrogenase from *Pyrobaculum islandicum* (*Pis*GDH). When expressed heterologously in *E. coli*, *Pis*GDH was not fully active despite its status as a soluble protein. However, when the recombinant enzyme was heated at 90 °C or exposed to 5 M urea, its activity increased to a level comparable to that of the native enzyme [13]. *Pis*GDH forms a hexameric structure, and heat-induced subunit rearrangement is considered essential for its activation. Therefore, we would expect to observe the expression of fully active *Pis*GDH in *Thermus* cells, which are cultured at a high temperature (70 °C). In this report, we describe the development of a new heterologous expression system for *T. thermophilus* HB27 in which supersaturated silica is used as the inducer. Furthermore, we demonstrate the expression of a sufficient amount of an active and soluble heterologous gene product. Our findings indicate the potential application of this system for the expression of thermostable enzymes at practical quantities.

## Results and discussion

### Construction of a silica-inducible expression vector and reporter plasmids

We previously reported that *Thermus* strains produced a specific Sip in the presence of supersaturated silica [12]. This Sip exhibited high homology with a Fe^3+^-binding ABC transporter that plays a role in Fe^3+^ uptake. The expression of Sip is thought to be a response to iron starvation stress, which can be induced by the addition of supersaturated negatively charged colloidal silica that captures Fe^3+^ ions. The ferric uptake regulator (Fur), a repressor of iron uptake-related gene expression, responds to low environmental iron levels and is released form promoter region to enhance downstream gene transcription. In an earlier study, the cultivation of *Thermus* cells in medium containing 10 mM silica led to an approximately tenfold higher Sip expression level relative to that observed in the absence of silica [11]. Therefore, we speculated that the *sip* promoter, which controls protein expression via a mechanism induced by silicic acid, could be utilized to express heterologous proteins in *Thermus*.

**Fig. 1:**
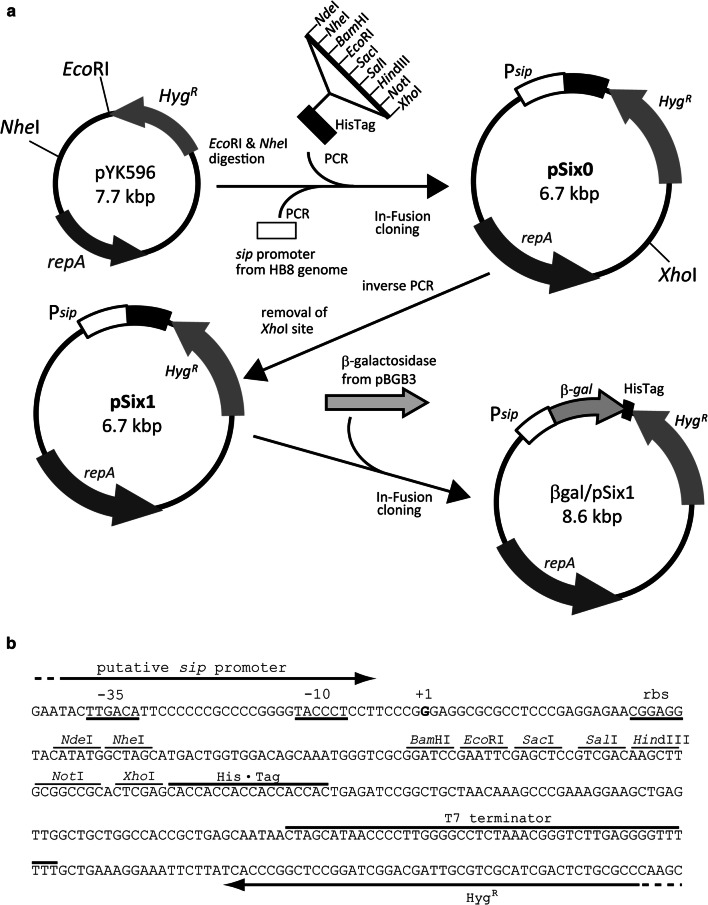
**a** Construction of the expression vector pSix1 for *Thermus* strains. The plasmid pYK596, which possesses a hygromycin resistance gene, was digested using *Eco*RI and *Nhe* I. Two PCR-amplified fragments, a multi-cloning site derived from pET21a, and a putative silica-inducible protein (*sip*) promoter region [12] were cloned into pYK596 using an In-Fusion cloning kit. The *Xho* I site on the pYK596 backbone was deleted by inverse PCR, and the resultant plasmid was named pSix1. To complete the Miller assay, a thermostable β-galactosidase gene from the *Thermus* sp. A4 was cloned downstream of the *sip* promoter of pSix1 to yield βgal/pSix1. **b** Sequence of pSix1. The − 35 and − 10 regions of the *sip* promoter are underlined. The experimentally determined transcription initiation site (+1) is indicated in bold, and the putative ribosome binding site (rbs) is underlined. Restriction sites in the multi-cloning site and 6× histidine tag sequences are indicated above the sequence

The construction of the plasmid DNA construct used in this study is illustrated in Fig. 1. We inserted a multi-cloning site (MCS) and a 600 bp fragment of a putative silica-inducible promoter region from *T. thermophilus* HB8 into the pYK596 plasmid, which contains a thermostable hygromycin resistance gene. Next, the *Xho* I site on the pYK596 backbone was eliminated by site-directed mutagenesis to yield the plasmid pSix1. To enable a reporter assay, a gene encoding a thermostable β-galactosidase derived from *Thermus* sp. A4 (βgal) was inserted into pSix1 at the *Nde* I and *Xho* I sites within the MCS. All the modified regions in this new βgal/pSix1 reporter plasmid were confirmed using an ABI 3130 genetic analyzer (Applied Biosciences, Foster City, CA, USA).

### Effect of supersaturated silica on the growth of *T. thermophilus*

As described above, the addition of silicic acid to *Thermus* cells may induce stress. Therefore, the effects of silicic acid on the growth of *Thermus* cells was monitored. Growth inhibition was observed in *T. thermophilus* HB27 cells harboring βgal/pSix1 after cultivation in medium containing various concentrations of silica, particularly those corresponding to supersaturation (> 5 mM at 70 °C; Additional file 1: Figure S1). Only slight cell growth inhibition was observed at 6.7 mM silica, whereas significant inhibition was observed at 10 mM silica, as indicated by an optical density at 660 nm (OD_660_) value that was reduced to nearly half the value observed at 0 mM silica. Although growth appeared to be impeded by silica-induced iron deficiency, the lack of a severe effect of exposure to 10 mM silica on the growth of wild-type *T. thermophilus* HB27 (data not shown) suggests that the observed growth inhibition may be attributable to the allocation of various energy resources toward protein expression rather than cell growth.

### Effect of the silicic acid concentration on β-galactosidase activity

β-Galactosidase is a convenient enzyme, as its activity can be quantitated precisely in liquid assays [14]. Consequently, it has been used widely as a reporter to monitor gene expression. Approximately 30 years ago, Koyama and co-workers first reported the use of a thermostable β-galactosidase gene as a reporter in *Thermus* strains [6]. Since then, several researchers have attempted to use β-galactosidase assays in studies of *Thermus* [8, 15]. However, wild-type *Thermus* cells exhibit a high level of background activity, and consequently it has been difficult to assess the precise level of promoter activity, particularly if the target promoter is relatively weak. To overcome this limitation, Fujita and co-workers recently developed a precise reporter assay system in which the background activity was reduced by disrupting all β-galactosidase genes and the phytoene synthase gene (*crtB*) in the host cells [16].

**Fig. 2:**
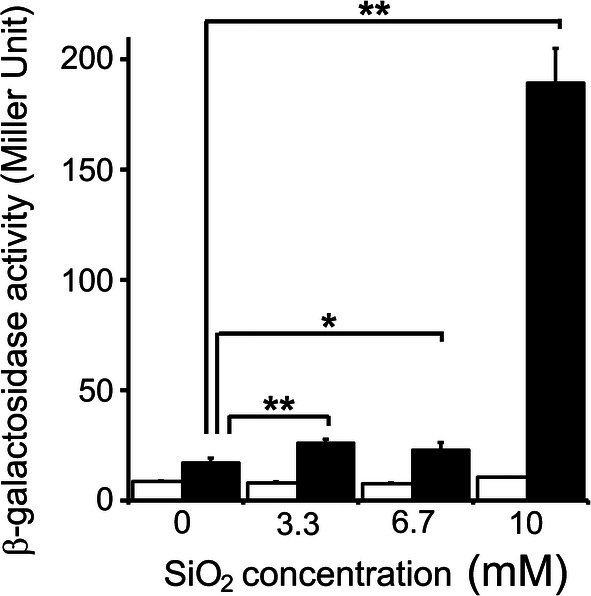
Induction of the *sip* promoter with different concentrations of silicic acid. A 5% suspension of pre-culture was inoculated in freshly prepared TM medium containing silicic acid and cultivated for 24 h. The β-galactosidase activity levels in culture are given in Miller units (MU). White bar: HB27 with pSix1 (empty vector), black bar: HB27 with βgal/pSix1. Values are expressed as means ± standard deviations (n = 3)

Despite those earlier findings, we selected wild-type HB27 as the host strain in our β-galactosidase assay because a previously reported qRT-PCR analysis revealed strong *sip* promoter activity (16-fold increase in response to 10 mM silica) [12]. As expected, exposure to 10 mM silica induced a sufficiently high level of β-galactosidase activity to enable a comparison between the induced and non-induced conditions. The β-galactosidase activity levels measured after a 24 h cultivation at various silica concentrations are indicated in Fig. 2. Notably, the β-galactosidase activity level was negligibly lower in non-induced condition than in cells under inducing conditions. HB27 cells with empty pSix1 vector exhibited an activity level < 10 Miller unit (MU), similar to that measured in HB27 cells without plasmid (data not shown). Relative to cells harboring pSix1, cells harboring the βgal/pSix1 plasmid exhibited slightly higher levels of activity (17–26 MU) when exposed to silica concentrations of 0–6.7 mM silica, suggesting leaky expression. However, when cultured in 10 mM silica, cells harboring βgal/pSix1 achieved β-galactosidase activity levels as high as 190 MU (11-fold higher than that observed at 0 mM silica), indicating strong *sip* promoter activity. On the other hand, β-galactosidase activity levels reached the plateau in higher silica concentration (> 10 mM). Moreover, higher concentrated silica solution was difficult to handle because dissolved silica easily precipitated during the cultivation. Accordingly, we set optimum silica concentration for induction to 10 mM.

Exposure to supersaturated silica indirectly causes iron starvation, leading to induction of the *sip* promoter. Therefore, we also tested direct iron starvation caused by iron chelators such as 2,2′-dipyridyl (DP) and 1,10- phenanthroline (phen). However, the addition of DP and phen (0.5–5 mM) did not have a notable effect on β-galactosidase expression (data not shown). This result implies that *Thermus* can utilize chemically masked iron species, such as siderophores [17]. Therefore, the addition of supersaturated silica is a superior method for initiating *sip* promoter-mediated transcription.

### Effect of medium exchange on gene expression

As described above, β-galactosidase activities were measured after a continuous exposure to silica from the beginning of the cultivation. However, induction during the middle or late exponential growth phase is generally much more advantageous for protein expression. Unlike isopropyl-β-d-1-thiogalactopyraoside (IPTG), highly concentrated silica is easily precipitated. Consequently, it is difficult to achieve an appropriate silicic acid concentration when using a stock solution. We opted for a medium exchange to achieve silica-mediated induction during the late exponential growth phase. Briefly, cells harboring βgal/pSix1 were cultivated in normal TM medium without silica until the OD_660_ value reached 0.6. The cells were then collected by centrifugation and inoculated in fresh medium containing 10 mM silica. The β-galactosidase activity in the cultures increased gradually along with the duration of silica exposure, reaching a plateau at 12 h. As shown in Additional file 1: Table S3, however, the activity levels after medium exchange were lower than those observed after uninterrupted cultivation in medium containing supersaturated silica. Although the maximum activity value achieved in a medium exchange culture was 3.6-fold higher than that observed before induction, this maximum activity was less than one-third of the value observed after an uninterrupted culture period in 10 mM silica. In a recent report, we similarly observed that medium exchange was ineffective in an *E. coli*-based protein expression system that utilizes silica as an inducer in advance [12]. As discussed in previous reports, this decrease in protein expression may be attributable to intracellular iron storage proteins. Because normal silica-free medium contains sufficient iron, cells grown in this medium can take up sufficient levels of iron to maintain growth and can store this element as bacterioferritin [18]. As these bacterioferritin molecules are then passed to daughter cells, several generations may be required to provoke iron starvation. Therefore, exposure to silica throughout the cultivation period resulted in much higher protein expression levels.

### Immunodetection of His-tagged β-galactosidase

**Fig. 3:**
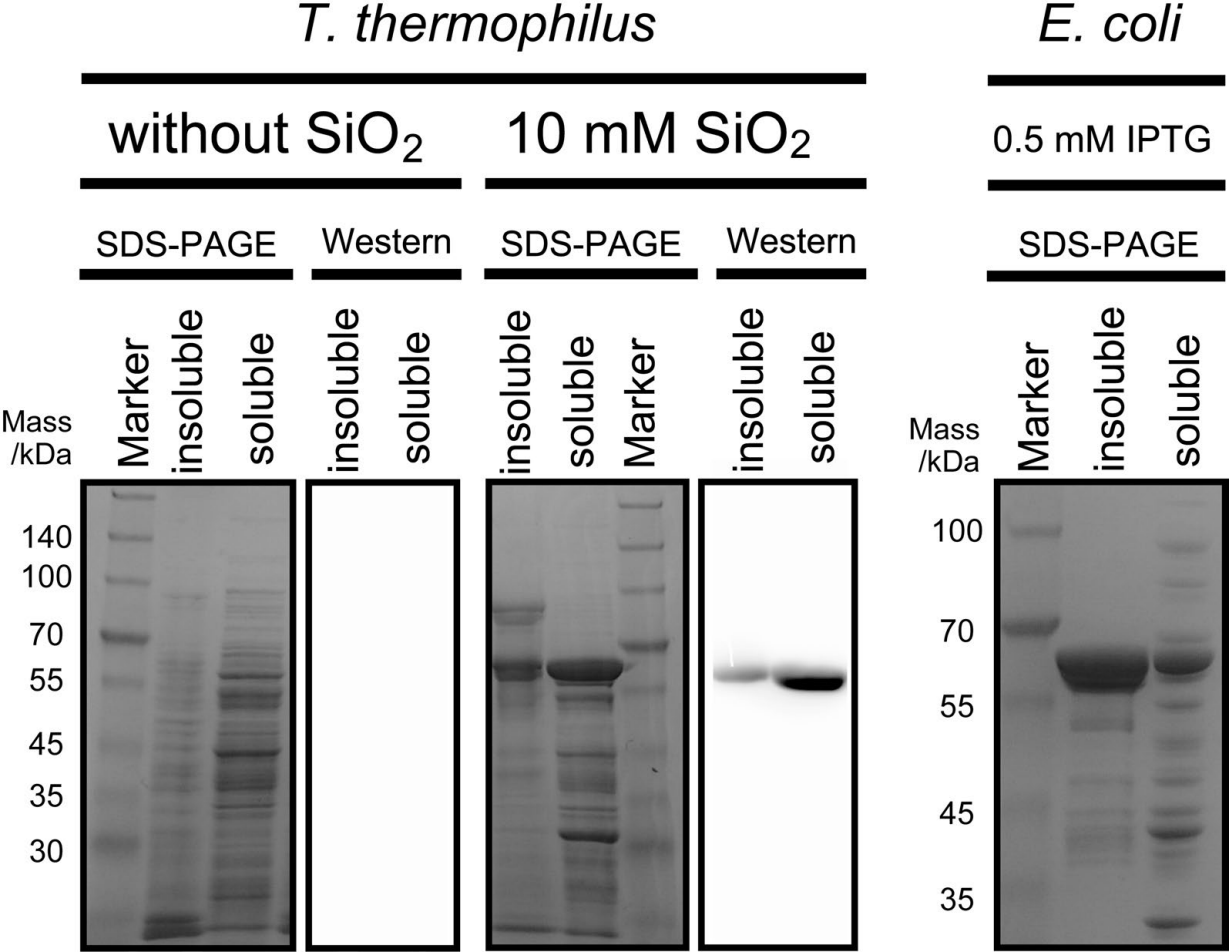
SDS-PAGE and Western blot analysis of recombinant proteins produced in *T. thermophilus* and *E. coli*. Left: Expression of His-tagged β-galactosidase in *T. thermophilus* HB27 in response to 0 and 10 mM silicic acid. Right: Expression of recombinant β-galactosidase in *E. coli*. Cells were harvested by centrifugation and disrupted by sonication. The soluble fraction was separated from insoluble fractions by centrifugation. Each lane contains 10 µg of total protein. His-tagged proteins were detected using anti-His-tag mAb-HRP-DirecT

We next examined whether our *T. thermophilus*-based heterologous expression system could be applied for practical use. In light of our previous analysis, β-galactosidase was induced by continuous exposure to 10 mM silica. After a 48-h cultivation, the cell extracts were subjected to SDS-PAGE and Western blotting (Fig. 3). In the absence of silicic acid, no protein bands corresponding to β-galactosidase were observed in either the insoluble or soluble fraction after an SDS-PAGE analysis, and no corresponding bands were detected by Western blotting. By contrast, the extracts of cells exposed to 10 mM silica produced clearly visible bands on SDS-PAGE. Bands corresponding to His-tagged β-galactosidases were also detected on Western blots. Particularly strong bands were detected in the soluble fraction, suggesting that the recombinant form of β-galactosidase expressed in *T. thermophilus* was correctly folded.

If heterologous proteins do not require post-translational modifications, *E. coli* is usually first selected to obtain enough material for both research and industrial applications. However, overexpressed proteins fail to reach a correct conformation and form insoluble aggregates of nonnative proteins called inclusion bodies [19]. Formation of inclusion bodies in *E. coli* is mainly due to rapid protein production during the forced expression, however, with respect to thermoenzymes, domain misfolding is also attributable to lower temperature than their natural hosts [5]. When we expressed recombinant β-galactosidase in *E. coli*, we found that most of the protein was expressed in the insoluble fraction, indicating the formation of inclusion bodies (right panel in Fig. 3). This discrepancy in protein production between the species may be attributable to the high temperature under which *T. thermophilus* is cultivated, as thermophilic enzymes occasionally require exposure to high temperatures to achieve correct folding into catalytically active forms [13, 20–22]. Our successful achievement of soluble protein expression in *T. thermophilus* suggests that our expression vector can serve as a useful genetic tool for the expression of thermostable gene products that would be insoluble when produced in *E. coli*.

Previously, Moreno and co-workers reported the achievement of heterologous gene expression in *T. thermophilus* at a practical level [7]. The system developed by these authors adopted the nitrate reductase operon (*nar* operon) in *T. thermophilus* HB8, which is strongly transcribed in response to nitrate under anoxic conditions [23]. Therefore, their system requires the specific host strain *T. thermophilus* HB27::*nar* (transplanted with the *nar* cluster from HB8) to enable anaerobic growth in the presence of nitrate [24]. By contrast, our system, which is driven by a silica-inducible promoter, does not require a genetically modified host strain because Sip induction is common among *Thermus* species and is basically controlled by ferric uptake regulators that are inherent in most microorganisms. These features may enhance the convenience of our system and its applicability to hundreds of other strains in the genus *Thermus*. These advantages of a silica-inducible promoter highlight the potential use of *T. thermophilus* as a host strain for the expression of thermostable proteins and other practical applications (e.g., microbial bioprocesses) that require relatively high temperatures.

#### Promoter deletion analysis

**Fig. 4:**
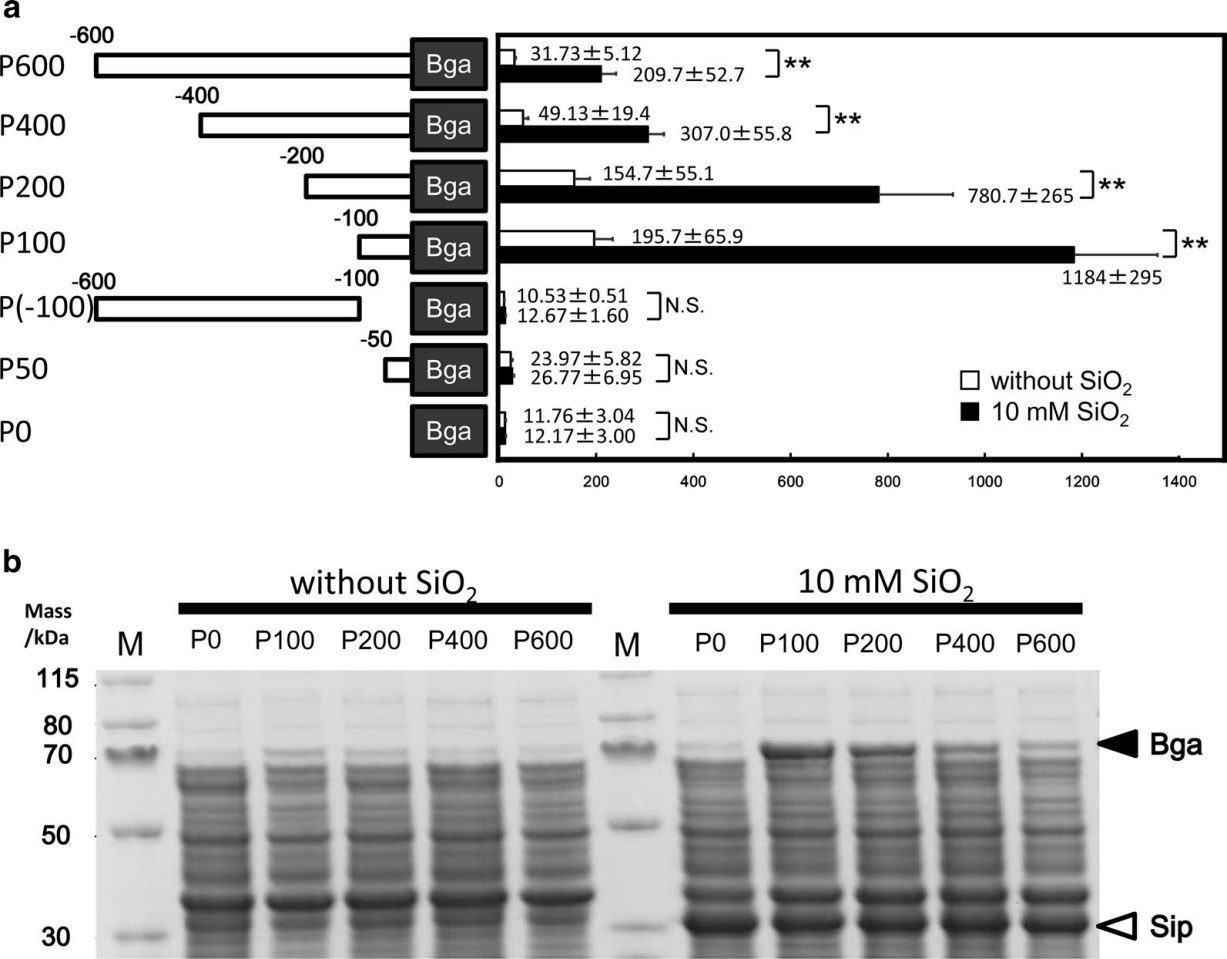
Promoter deletion analysis using a β-galactosidase reporter system. **a** Schematic figure of the promoter region and β-galactosidase activity in the presence or absence of supersaturated silica. The β-galactosidase activity levels are presented as the mean ± standard deviations of three independent experiments and are shown in Miller units. The white and black bars indicate the activity levels in response to 0 and 10 mM silica, respectively. **b** Image of an SDS-PAGE analysis of expressed proteins from plasmids containing promoter deletion mutants. Cells were harvested from 24 h cultures and disrupted by sonication, after which the soluble fractions were subjected to 12% SDS-PAGE. The black and white arrowheads indicate the bands corresponding to β-galactosidase and silica-induced protein, respectively

Next, deletion mutants of βgal/pSix1 were constructed to determine the minimum region of the active promoter. We found that a 100 bp region upstream of the *sip* coding sequence (CDS) exhibited significant promoter activity, whereas a 50 bp upstream region exhibited no activity. Also the upstream region 600 to 100 bp distal from the *sip* CDS did not appear to be important for promoter activity. We previously determined transcriptional start site (TSS) of the *sip* gene under supersaturated silica condition in *T. thermophilus* strain HB8 [12]. Although we used *T. thermophilus* strain HB27 as an expression host, putative *sip* promoter from strain HB8 was introduced into βgal/pSix1. DNA sequence of this promoter fragment from HB8 is almost identical with that of HB27 (Additional file 1: Figure S2), thus, it seems to function in HB27 cells in the same manner. Therefore, the essential *sip* promoter region appeared to be contained within the region 100 to 50 bp upstream of the start of the *sip* CDS (Fig. 4a). In our earlier reports, it was experimentally confirmed that a Fur (TTHA0344 product) interacted with the region within 150 bp upstream of *sip* CDS, however, Fur binding consensus sequence seen in other bacteria was not found in *T. thermophilus* [12]. Although the sequence of Fur binding site in *T. thermophilus* remains unclear, 100 bp upstream region of the *sip* CDS can contain at least one promoter region and one Fur binding site.

Interestingly, the 100-bp promoter yielded a β-galactosidase activity level of approximately 1200 MU, which was 6 times higher than the level achieved with the 600-bp promoter region. This phenomenon was also confirmed by SDS-PAGE, which revealed that the shorter promoter region yielded a stronger thermostable β-galactosidase band (Fig. 4b). Our results suggest that the 100-bp *sip* promoter region yielded much more efficient expression of the recombinant protein than the original expression vector pSix1. Consequently, we truncated the promoter region of pSix1 to 100 bp to yield pSix3.

Mini-prep extraction revealed that pSix1 produced a lower DNA yield than the original plasmid, pYK596, suggesting an issue of stability with the former construct. Because the *sip* promoter regions in the plasmid DNA (derived from *T. thermophiles* HB8) and in the host chromosomal DNA (*T. thermophiles* HB27) are so similar (99%), we suspected that homologous recombination might have occurred. Specifically, a single-crossover homologous recombination event would have led to the insertion of plasmid DNA into the chromosomal DNA. Additional file 1: Figure S3_A presents a schematic of the gene arrangements of normal chromosomal DNA, pSix1, and chromosomal DNA in a single-crossover mutant. To confirm the single-crossover homologous recombination event, total DNA from various transformants harboring the plasmids with different promoter regions were extracted and then subjected to PCR amplification analysis. As shown in Additional file 1: Figure S3_B, βgal/pSix1 yielded a band that corresponded to the single-crossover region between chromosomal and plasmid DNA. No crossover associated bands were observed with any other plasmids, including pSix3, indicating the stable maintenance of these plasmids in the host cells.

In our experiments, the 100-bp promoter yielded the highest expression level. However, this promoter was also associated with a higher leaky expression level than the long promoter. We speculated that this might be attributable to a competition effect of Fur protein, because there are more Fur binding sites on plasmid DNA competing for the same amount of Fur proteins. In a previous report, the estimated plasmid copy number of pTT8 (the precursor plasmid of pYK596) relative to chromosomal DNA was eight [25, 26]. This means that there are also up to eight times more Fur binding sites available for the same amount of Fur which perhaps would explain the higher leaky expression. Further modifications, such as an insertion of the *fur* gene into the expression vector, might be needed to achieve more strict expression control.

#### Homologous expression of β-galactosidase and heterologous expression of *pis*GDH via pSix3

**Fig. 5:**
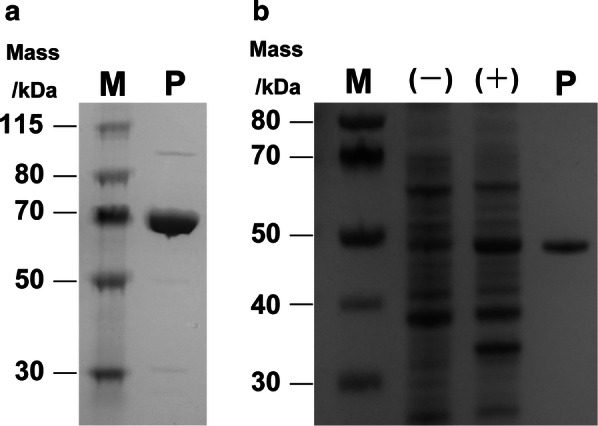
Purification of thermostable β-galactosidase and *pis*GDH expressed in *Thermus* cells. **a** Purification of thermostable β-galactosidase. **b** Purification of *pis*GDH. (−); 0 mM silica, (+); 10 mM silica. Lane P indicates the fraction purified via nickel-affinity chromatography on a Ni-NTA column. Lane M contains the molecular mass marker

We determined that pSix3 was most suitable for protein expression in *T. thermophilus* HB27. Subsequently, we successfully expressed β-galactosidase from *Thermus* sp. A4 as shown in Fig. 4b. His-tagged β-galactosidase was purified using immobilized nickel-affinity chromatography, as confirmed by the appearance of a single band on SDS-PAGE (Fig. 5a), and yielded 27 mg/L of culture. This expression level was the highest achieved using this *T. thermophilus*-based system. However, this result should be considered homologous expression because the target gene was β-galactosidase from a *Thermus* strain.

To confirm the ability of this system to induce heterologous expression, we attempted to express glutamate dehydrogenase from the hyperthermophilic archaeon *Pyrobaculum islandicum* (*pis*GDH), which requires heat treatment to reach a fully activated form. First, the original *pis*GDH gene was cloned into the pSix3 vector. However, this plasmid did not yield protein expression in *Thermus* cells, which we attributed to a codon bias between *Thermus* and *Pyrobaculum*. We then codon-optimized a *pis*GDH gene fragment for *Thermus* via artificial synthesis, which enabled the successful expression of *pis*GDH in *Thermus* cells (Fig. 5b). After purification via affinity chromatography, this system yielded 9.5 mg *pis*GDH/L of culture. As expected, *Thermus* expressed a fully active form of *pis*GDH without any further treatment, thus emphasizing the superiority of this expression system for thermostable enzyme production. A specific activity analysis yielded a value of 4.90 µmol/min/mg for the enzymes expressed in *Thermus*, which was nearly identical to that of native *pis*GDH (3.51 µmol/min/mg).

Our findings support our initial conjecture that fully activated thermostable enzymes could be expressed in *Thermus* cells cultured at high temperatures. However, we note that our original failure to express *pis*GDH from the original (i.e., not optimized for *Thermus*) sequence cannot be ignored. *Thermus* species have a high GC content (~ 70%) and therefore a very different codon usage pattern from that of other organisms. A new host strain containing tRNAs for codons that are rarely used in *Thermus* might be required to achieve universal translation.

## Conclusion

We have reported the development of a silica-inducible promoter-based system to achieve the homologous and heterologous expression of thermostable genes in *Thermus thermophilus*. Notably, we successfully expressed soluble and thermostable *Thermus* β-galactosidase in *Thermus* cells and demonstrated that this system was more efficient than an *E. coli*-based expression system. We further optimized the promoter region via a promoter deletion assay and ultimately achieved a β-galactosidase protein yield of 27 mg/L of culture, the highest value reported for a *Thermus* expression system to date. Moreover, the successful use of our novel system to express the heterologous *pis*GDH supports our hypothesis that a *Thermus*-based hot expression system would enhance the production of fully active thermostable enzymes. We conclude that our novel expression system represents a substantial contribution with respect to enzymatic research and, potentially, industrial applications.

## Materials and methods

### Strains and growth conditions

*Thermus thermophilus* strains HB8 (ATCC 27634) and HB27 (ATCC BAA-163) were grown in *Thermus* medium (TM medium) composed of 4.0% (w/v) BBL™ Polypeptone™ Peptone (Becton–Dickinson, United States), 2.0% (w/v) Bacto™ Yeast Extract (Becton–Dickinson), 1.0% (w/v) sodium chloride (Nacalai tesque, Japan) and 0.1% Castenholz’ basal salt solution under strong aeration in baffled flask (i.e., rotation at 180 rpm) at 70 °C [27, 28]. TM medium containing silicic acid was prepared using a 1000 ppm sodium orthosilicate stock solution in 10 mM NaOH. Prior to use, the medium was pH-adjusted to 7.2 with HCl and autoclaved [11]. Hygromycin (100 µg/mL) was added to liquid or agar medium as needed.

### Plasmid constructions and overexpression of β-galactosidase

The plasmids and primers used in this study are listed in Additional file 1: Tables S1 and S2. The *Thermus* cloning vector pYK596 (7733 bp), which carries a thermostable hygromycin resistance gene, was kindly provided by Dr. Koyama (National Institute of Advanced Industrial Science and Technology, Japan, unpublished). The pBGB3 plasmid containing thermostable β-galactosidase was kindly provided by Dr. Motoshima (Yotsuba Milk Products Co., Ltd, Japan) [29]. The silica-inducible expression vector pSix1 and reporter plasmid βgal/pSix1 were constructed as shown in Fig. 1. The putative *sip* promoter region (600 bp) was amplified from the chromosomal DNA of *T. thermophilus* HB8 (NCBI Accession Number: AP008226) using LA *Taq* DNA polymerase (TaKaRa-bio Inc., Japan) with the primers IFp6SF and IFp6SR. The MCS containing a C-terminus His-tag was amplified from pET21a (Novagen, United States) using the primers IFmcsF and IFmcsR. These amplified fragments were cloned into pYK596 at the *Nhe* I (2852) and *Eco* RI (992) sites using the In-Fusion HD cloning kit (TaKaRa-bio). The resultant plasmid was named pSix0 (plasmid for silica-inducible expression, 6700 bp). The *Xho* I site at position 1975 nt was removed by site-directed mutagenesis with the primers pSixdexhoF and pSixdexhoR, and the resultant plasmid was named pSix1 (DDBJ Accession number: LC428096).

For the β-galactosidase reporter assay, a thermostable β-galactosidase gene of *Thermus* sp. A4 was PCR-amplified from pBGB3 using the primers IFbgal-sixF and IFbgal-sixR. The PCR product was then cloned into linearized pSix1 amplified by inverse PCR with the primers pSix-invL and pSix-invR using the In-Fusion HD cloning kit. The resultant plasmid was named βgal/pSix1.

To achieve the overexpression of thermostable β-galactosidase in *E. coli*, the β-galactosidase gene of *Thermus* sp. A4 was PCR-amplified from pBGB3 using the primers IFbgal-pETF and IFbgal-pETR. The PCR product was cloned into linearized pET21a (Novagen) after *Nde* I and *Xho* I digestion, using the In-Fusion HD cloning kit. The resultant plasmid, βgal/pET21a, was used to transform *E. coli* Rosetta™2 (DE3) competent cells (Merck Millipore, United States). A single colony of freshly transformed cells was inoculated into LB medium (1.0% (w/v) tryptone (Nacalai tesque), 0.5% (w/v) yeast extract (Nacalai tesque), and 1.0% (w/v) sodium chloride) containing 50 µg/mL ampicillin and 50 µg/mL chloramphenicol.

### Expression of β-galactosidase in *T. thermophilus* and *E. coli*

The recombinant β-galactosidases were expressed in both *E. coli* and *T. thermophilus* HB27. For expression in *E. coli*, Rosetta™2 (DE3) competent cells (Merck Millipore) was transformed with the recombinant plasmid βgal/pET21a. Protein expression was induced by adding 0.5 mM of isopropyl-β-d-1-thiogalactopyranoside (IPTG) to exponentially growing cells (OD_660_ = 0.6), followed by further cell growth for 3 h. The cells were then collected by centrifugation and lysed in an appropriate volume of BugBuster^®^ HT Protein Extraction Reagent (Merck Millipore). The soluble and insoluble fractions were subjected to 10% SDS-PAGE. For expression in *T. thermophilus*, 5 mL of an overnight culture of strain HB27 harboring the plasmid βgal/pSix1 were inoculated into 100 mL of freshly prepared TM medium containing 10 mM silicic acid and cultivated at 70 °C. The cells were then harvested and lysed using BugBuster^®^ HT Protein Extraction Reagent. The soluble and insoluble fractions were subjected to 10% SDS-PAGE. Protein concentration was determined using a Quant-iT™ Protein Assay Kit (Invitrogen, United States).

The expression of His-tagged recombinant β-galactosidase was confirmed by Western blotting. Electrophoresed proteins were transblotted onto a polyvinylidene difluoride (PVDF) membrane for 30 min at 150 mA in transfer buffer containing 25 mM Tris (pH 8.3), 192 mM glycine, and 20% (v/v) methanol. The membrane was then incubated in blocking buffer [5% skimmed milk in 1× Tris buffered saline (TBS; 5 mM Tris, 138 mM NaCl, and 2.7 mM KCl)] for 1 h at room temperature, and then incubated with 1/5000 diluted anti-His-tag mAb-HRP-DirecT (Medical and Biological Laboratories Co., Ltd. Japan) in blocking buffer for 30 min. After three washes with TBS-T (0.05% Tween 20 (Nacalai tesque, Japan) in 1× TBS), the membrane was incubated with ImmunoStar Zeta reagent (WAKO, Japan). Specific labeled proteins were visualized using an AE-9300 Ez-Capture MG imaging system (ATTO, Japan).

### β-Galactosidase assays

β-Galactosidase assays were performed as previously described [30], with slight modification. Briefly, after measuring the OD_600_, 1 mL aliquots of the cultures were centrifuged at 10,000× for 3 min. Cell pellets were resuspended in 100 µL of permeabilization solution (100 mM Na_2_HPO_4_, 20 mM KCl, 2 mM MgSO_4_, 0.8 mg/mL hexadecyltrimethylammonium bromide, 0.4 mg/mL sodium deoxycholate, and 5.4 µL/mL β-mercaptoethanol) and maintained for 30 min at 70 °C. Next, 900 µL of substrate mixture [60 mM Na_2_HPO_4_, 40 mM NaH_2_PO_4_, 1 mg/mL *o*-nitrophenyl-β-d-galactoside (ONPG), and 2.7 µL/mL β-mercaptoethanol] prewarmed to 70 °C was added to initiate the reaction. After sufficient color had developed (15–60 min), the reactions were terminated by the addition of 1 mL of 1 M Na_2_CO_3_. The reaction mixtures were then centrifuged at 10,000×*g* for 5 min, and the A_420_ values of the supernatants were recorded. Enzyme activities were expressed in Miller units (MU) and were calculated as the A_420_/reaction time (min)/culture volume assayed (mL)/OD_600_. In all experiments, activity was measured in three independent assays.

### Promoter deletion analysis

To identify the minimal region of the *sip* promoter, a series of promoter deletion fragments were fused to the thermostable β-galactosidase gene from *Thermus* sp. A4. Specifically, the promoter region was truncated by inverse PCR of βgal/pSix1 with the primers listed in Additional file 1: Table S2. *T. thermophilus* HB27 was transformed with these plasmids and then cultivated in TM medium containing 10 mM silicic acid at 70 °C for 24 h. The β-galactosidase activity levels were determined as described above. Consequently, the 100-bp *sip* promoter region was identified as most effective. Accordingly, an expression vector containing this 100-bp *sip* promoter region and MCS was created in the same way. The resultant plasmid was named pSix3 and has been deposited in the DDBJ database (Accession number: LC504201).

### Statistical analysis

The level of expression of β-galactosidase in induced cells was compared to the level of expression in uninduced cells. The data were presented as the mean ± SE. Three independent samples were analyzed using Student’s t test using Microsoft Excel^®^. Statistical differences were considered significant if the p value was less than 0.05 (denoted by stars *p < 0.05; **p < 0.005).

### Expression of β-galactosidase from pSix3

A thermostable β-galactosidase gene was cloned into pSix3 as described earlier in this section. His-tagged β-galactosidase was expressed in TM medium containing 10 mM silicic acid for 48 h. Subsequently, the cells were collected by centrifugation, resuspended in IMAC30 Buffer [20 mM Tris–HCl (pH 8.0), 300 mM NaCl, 30 mM imidazole], and disrupted using an ultrasonic disrupter. The cell debris was removed by centrifugation, and the supernatant was applied to a nickel-nitrilotriacetic acid (Ni-NTA) agarose column (Qiagen, Germany) and eluted with IMAC250 Buffer [20 mM Tris–HCl (pH 8.0), 300 mM NaCl, 250 mM imidazole]. The purified fraction was desalted and concentrated by ultrafiltration (Merck Millipore). The final protein concentration was determined using a Quant-iT™ Protein Assay Kit (Invitrogen).

### Heterologous expression of *Pis*GDH in *Thermus* spp.

The *Pyrobaculum islandicum* glutamate dehydrogenase gene sequence was optimized for expression in *T. thermophilus* HB27 by substituting rare codons found in the latter organism (Codon Usage Database: https://www.kazusa.or.jp/codon/). The original and optimized *pis*GDH sequences are listed in Additional file 1: Table S4. The optimized *pis*GDH sequence was synthesized artificially as a gBlocks^®^ Gene Fragment (Integrated DNA Technologies, United States). This fragment was amplified by PCR and cloned into pSix3 using the In-Fusion HD cloning kit. Fifty milliliters of a pre-culture of this transformant were inoculated into 1 L of freshly prepared TM medium containing 10 mM silica and cultivated for 48 h.

His-tagged *pis*GDH was purified as follows. First, the cells were harvested and resuspended in an appropriate volume of Binding/Wash Buffer [10 mM potassium phosphate buffer (pH 7.2), 300 mM NaCl, 20 mM imidazole, 10% glycerol] and disrupted by ultrasonication. After removing the cell debris by centrifugation, the supernatant was applied to a Ni-NTA agarose column (Qiagen). The target protein was eluted with 300 mM imidazole, and the eluates were dialyzed against Standard Buffer [10 mM potassium phosphate buffer (pH 7.2), 1 mM EDTA, 0.1 mM DTT, 10% glycerol]. Samples for the activity assay were further purified by size exclusion chromatography. The protein concentrations were determined using a Bio-Rad Protein Assay (Bio-Rad). The enzymatic activity of *pis*GDH was assayed spectrophotometrically by monitoring the increase of NADH at 340 nm [31].

## Supplementary information

**Additional file 1: Table S1**. Plasmids used in this study. **Table S2**. Primers used in this study. **Figure S1**. Effect of silicic acid on the growth of *Thermus thermophilus* HB27 cells harboring the βgal/pSix1 plasmid. **Table S3.** β-Galactosidase activity after medium exchange. **Figure S2.** Comparison of putative sip promoter region between *T. thermophilus* HB8 and HB27 **Figure S3**. Homologous recombination between chromosomal DNA and plasmid DNA. **Table S4.** Sequence of *pis*GDH for expression in *Thermus thermophilus*.

## Data Availability

The recombinant strains described in this work will be made available upon request to the corresponding author. Data sharing is not applicable to this article, as no datasets were generated or analyzed during this study.

## Abbreviations

*pis*GDH: Glutamate dehydrogenase from *Pyrobaculum islandicum*
ABC transporter: ATP-binding cassette transporter
Sip: Silica-induced protein
MCS: Multi-cloning site
OD: Optical density
qRT-PCR: Quantitative reverse transcription polymerase chain reaction
MU: Miller unit
DP: 2,2′-dipyridyl, phen; 1,10-phenanthroline
IPTG: Isopropyl-β-d-1-thiogalactopyranoside
SDS-PAGE: Sodium dodecyl sulfate-polyacrylamide gel electrophoresis
CDS: Coding sequence
PVDF: Polyvinylidene difluoride
ONPG: *o*-nitrophenyl-β-d-galactoside
IMAC: Immobilized metal affinity chromatography
NADH: Nicotinamide Adenine Dinucleotide − Hydrogen
EDTA: Ethylenediaminetetraacetic acid
DTT: Dithiothreitol
