# Supplementary material for: Development of a new gene expression vector for Thermus thermophilus using a silica-inducible promoter
Source: Microb Cell Fact. 2020 Jun 8;19:126. doi: 10.1186/s12934-020-01385-2 (PMC7282064; doi:10.1186/s12934-020-01385-2)
Supplement: Supplementary file 1 — Additional file 1: Table S1. Plasmids used in this study. Table S2. Primers used in this study. Figure S1. Effect of silicic acid on the growth of Thermus thermophilus HB27 cells harboring the βgal/pSix1 plasmid. Table S3. β-Galactosidase activity after medium exchange. Figure S2. Comparison of putative sip promoter region between T. thermophilus HB8 and HB27 Figure S3. Homologous recombination between chromosomal DNA and plasmid DNA. Table S4. Sequence of pisGDH for expression in Thermus thermophilus. [file 12934_2020_1385_MOESM1_ESM.docx]

**Table S1 Plasmids used in this study**

| **plasmid** | **characteristic** | **Source** |
| --- | --- | --- |
| **pYK596** | *Thermus* vector with thermostable hygromycin resistant gene derived from pTT8 in *T. thermophilus* HB8 | Gift from Dr. Koyama |
| **pBGB3** | pUC119-based plasmid carrying thermostable β-galactosidase from *Thermus* sp. A4. | (Ohtsu et al 1998) |
| **pSix0** | pYK596 derived expression vector with 600 bp *sip* promoter and multi-cloning site but still has additional *Xho*I site on its backbone | This study |
| **pSix1** | *Xho*I site on its backbone has been eliminated from pSix0 | This study (LC428096) |
| **bgal/pSix1** | pSix1 carrying thermostable β-galactosidase | This study |
| **pSix3** | pYK596 derived expression vector with 100 bp *sip* promoter and multi-cloning site | This study (LC504201) |

**Table S2 Primers used in this study**

| primers | sequences | purpose |
| --- | --- | --- |
| IF-p6S-F | 5’-AATCCTGGACGCTAGGGGCCAAGGGGAAGGGCCTAA-3’ | Cloning of *sip* promoter into pYK596 |
| IF-p6S-R | 5’-CATATGTACCTCCGTTCTCCTCGGGAGGC-3’ | Cloning of *sip* promoter into pYK596 |
| IF-mcs-F | 5’-ACGGAGGTACATATGGCTAGCATGACTGGTGGACAG-3’ | Cloning of multi-cloning site into pYK596 |
| IF-mcs-R | 5’-CGGGTGATAAGAATTTCCTTTCAGCAAAAAACCCCT-3’ | Cloning of multi-cloning site into pYK596 |
| pSixdexhoF | 5’-CAGGGGGAAAATCAGTG-3’ | Deletion of *Xho*I site from pSix0 |
| pSixdexhoR | 5’-GAGGACCGCCCCTAAAAC-3’ | Deletion of *Xho*I site from pSix0 |
| pSix-invL | 5’-ATGTACCTCCGTTCTCCTCGGGAGGCGCGCC-3’ | Amplification of pSix1 by inverse PCR |
| pSix-invR | 5’-CACCACCACCACCACCACTGAGATCCGGCTGCTAAC-3’ | Amplification of pSix1 by inverse PCR |
| IF-bgal-sixF | 5’-GAGAACGGAGGTACATATGCTCGGCGTTTGCTATTACCCCGAAC-3’ | Cloning of β-galactosidase gene into pSix1 |
| IF-bgal-sixR | 5’-GGTGGTGGTGGTGGTGTGCCTCCTCCCAGACGGCGAGGTCATAAG-3’ | Cloning of β-galactosidase gene into pSix1 |
| IF-bgal-pETF | 5’-AAGGAGATATACATATGCTCGGCGTTTGCTATTACC-3’ | Cloning of β-galactosidase gene into pET21a |
| IF-bgal-pETR | 5’-GGTGGTGGTGCTCGAGTGCCTCCTCCTCCCAGACGCC-3’ | Cloning of β-galactosidase gene into pET21a |
| pSix-DM400-fw | 5’-AAGGCGGCGGCCTTGGCCCGGGCCTTTGAC-3’ | Deletion mutant of bgal/pSix1 (P400) |
| pSix-DM200-fw | 5’-CCACACCCTAAACCCCGTCCCCCTGGTCTA-3’ | Deletion mutant of bgal/pSix1 (P200) |
| pSix-DM100-fw | 5’-TCTAAAACCAATAAATCCGACCGGAATAC-3’ | Deletion mutant of bgal/pSix1 (P100) |
| pSix-DM50-fw | 5’-TACCCTCCTTCCCGGGAGGCGCGCC-3’ | Deletion mutant of bgal/pSix1 (P50) |
| pSix-DM0-fw | 5’-ATGCTCGGCGTTTGCTATTACCCCGAACAC -3’ | Deletion mutant of bgal/pSix1 (P0) |
| pSix-DM-rv | 5’-CTAGCGTCCAGGATTTCAATCCCCAGGAGC-3’ | Deletion mutant of bgal/pSix1 |
| pSix-DM(-100)-fw | 5’-ATGCTCGGCGTTTGCTATTACCCCGAACAC -3’ | Deletion mutant of bgal/pSix1 (P(-100)) |
| pSix-DM(-100)-rv | 5’-GTATTCCGGTCGGATTTATTGGTTTTAGA-3’ | Deletion mutant of bgal/pSix1 (P(-100)) |
| pSix-inv-L | 5’-ATGTACCTCCGTTCTCCTCGGGAGGCGCGCC-3’ | Amplification of pSix vectors by inverse PCR |
| pSix-inv-R | 5’-CACCACCACCACCACCACTGAGATCCGGCTGCTAA-3’ | Amplification of pSix vectors by inverse PCR |
| GDH-pSix-fw | 5’-AGAACGGAGGTACATATGGAAAGGACCGG-3’ | Cloning of *pis*GDH gene into pSix3 |
| GDH-pSix-rv | 5’-TGGTGGTGGTGCTCGAGGATCCAG-3’ | Cloning of *pis*GDH gene into pSix3 |
| chr-sip-fw | 5’-TTGGAGGACCTCTTCCCCTTCCTCC-3’ | Check for homologous recombination of plasmid |
| int-sip-rv | 5’-TGACCAAGGGTTCCACCAGGCTCTG-3’ | Check for homologous recombination of plasmid |
| pls-sip-fw | 5’-AGGGAAGGCTCCTGGGGATTGAAATCC-3’ | Check for homologous recombination of plasmid |
| hyg-fw | 5’-ATGAAAAAGCCTGAACTCACCGTGACG-3’ | Check for homologous recombination of plasmid |
| hyg-rv | 5’-TCACCCGGCTCCGGATCGGACG-3’ | Check for homologous recombination of plasmid |





**Figure S1 Effect of silicic acid on growth of *T. thermophilus* HB27 cells carrying βgal/pSix1.**

The growth curve in the absence of silicic acid is indicated with the dotted line and open circle plots. Closed symbols show the growth curve in the presence of silicic acid. Closed squares ■, 3.3 mM; closed triangles ▲; 6.7 mM; closed circles ●; 10 mM. Values are expressed as mean ± standard deviation (SD) of three independent experiments.

**Table S3 β-galactosidase activity after medium exchange**

| Time after medium exchange (h) | β-galactosidase activity (MU) | |
| --- | --- | --- |
|  | empty vector | βgal/pSix1 |
| 0 | 9.6 ± 0.8 | 17.2 ± 2.1 |
| 3 | 22.0 ± 5.7 | 38.9 ± 1.7 |
| 6 | 18.4 ± 4.9 | 43.7 ± 2.9 |
| 12 | 10.9 ± 0.9 | 61.7 ± 1.9 |
| 24 | 19.0 ± 1.0 | 43.6 ± 2.7 |

Cells were cultivated in TM medium without silicic acid until OD_660_ reached 0.6. Cells were then collected and re-suspended in freshly prepared TM medium with 10 mM silicic acid (time = 0 h). β-galactosidase activities are indicated as the mean ± standard deviation of three independent experiments.

HB8 GGGCCAAGGGGAAGGGCCTAAGGGTCCTCCACGCCAACGGCTACCGCCCGGACTACCTCAGGCGGGCCACGGAAGGGAAAAGGCTCCTCCTCTCCGCCTTCGCCCAGGCGGCCCGCCTCG

HB27 GGGCCAAGGGGAAGGGCCTAAAGGTCCTCCACGCCAACGGCTACCGCCCGGACTACCTCAGGCGGGCCACGGAAGGGAAAAGGCTCCTCCTTTCCGCCTTCGCCCAGGCGGCCCGCCTCG

HB8 CGGGCCTTCCCCTCCTCCCCCTGGGCCACCCCCTGGCCCTTCTCCCCGGCTTCTGGGAGGACCCCTACGGGGCGGGGGCCAAGGCGGCGGCCTTGGCCCGGGCCTTTGACCTCGTGGTCC

HB27 CGGGCCTTCCCCTCCTCCCCCTGGGCCACCCCCTGGCCCTTCCCCCCGGCTTCTGGGAGGACCCCTACGGGGCGGGGGCCAAGGCCGCCGCCTTGGCCCGGGCCTTTGACCTCGTGGTCC

HB8 TGGAGTACTGGGCCCTGGACCTCGCCGCCCACCGCCACCCCGAAAGCCTCCCCGACCGCTTCCGGGAGCTCACCCTTTTCCTCCAGGGCTTCCTCGCCGAAGGCGGCGTCCTCCTCCTGG

HB27 TGGAGTACTGGGCCCTGGACCTCGCCGCCCACCGCCACCCCGAAAGCCTCCCCGACCGCTTCCGGGAGCTCACCCTTTTCCTCCAGGGCTTCCTCGCCGAAGGCGGCGTCCTCCTCCTGG

HB8 CCTCGGACCACGGCAACGCCGAGGAGCCCTGGCACCCCCGCCACACCCTAAACCCCGTCCCCCTGGTCTACACCGAGGAGGCCCCGCCCCCACCGGAAGACCTCACGGGCGTCTTCCCCT

HB27 CCTCGGACCACGGCAACGCCGAGGAGCCCTGGCACCCCCACCACACCCTAAACCCCGTTCCCCTGGTCTACACCGAGGAAGCCCCGCCCCCACCGGAAGACCTCACGGGCGTCTTTCCCT

HB8 GGTTGCGAACGATTATCACTTCTAAAACCAATAAATCCGACCGGAATACTTGACATTCCCCCCGCCCCGGGGTACCCTCCTTCCCG**G**GAGGCGCGCCTCCCGAGGAGAACGGAGGTA

HB27 GGTTGCGAACGATTATCACTTCCAAAACCAATAAATCCGACCGGAATACTTGACATTTTCTCCACCCCGGGGTACCCTCCTTCCCGGGAGGCGCGCCTCCCGAGGAGAACGGAGGCA

**Figure S2 Comparison of putative *sip* promoter region between *T. thermophilus* HB8 and HB27**

Gray shading indicates the identical bases both in HB8 and HB27. Black shading indicates the transcription start site (TSS) under supersaturated silica condition determined in our previous report [12]. Enclosing characters show the putative -35 and -10 region of this promoter.





**Figure S3 Homologous recombination between chromosomal DNA and plasmid DNA**

A) Schematic figure of gene arrangement around *sip* promoter. Both chromosomal DNA and plasmid DNA (pSix1) possess similar *sip* promoter region, thus, single-crossover recombination would occur. Arrowheads indicate the primer binding site for the PCR to verify the replacement of genes. Primer sequences are listed on Table S1. chr-sip-fw and int-sip-rv amplify the *sip* upstream region on chromosomal DNA (fragment *g*; 920 bp), and hyg-fw and hyg-rv amplify the hygromycin resistance gene on plasmid DNA or replaced chromosome (fragment *p*; 909 bp), and the combination of pls-sipfw and int-sip-rv give the fragment of cross region between chromosome and plasmid (fragment *x*; 755 bp for pSix1, 255 bp for pSix3). B) Verification of the replacement of *sip* promoter region. bgal/pSix3 did not give the amplified band of cross region but bgal/pSix1 did, indicating single-crossover replacement occurred with long promoter region. This may cause low expression level with longer promoter due to plasmid instability.

**Table S4 *pis*GDH sequence for expression in *T. thermophilus***

| original |  | ATG GAG AGG ACA GGG TTT TTA GAG TAT GTG CTT AAC TAT GTA AAA AAA GGC GTA GAA TTA GGC GGA TTT CCC GAA GAT TTC TAT AAA ATC TTG TCT AGG CCT |
| --- | --- | --- |
| optimized |  | ATG GAG CGG ACC GGG TTC CTC GAG TAC GTG CTC AAC TAC GTG AAG AAG GGG GTG GAG CTC GGG GGG TTC CCC GAG GAC TTC TAC AAG ATC CTC TCC CGG CCC |
| amino acids | 001 | **Met Glu Arg Thr Gly Phe Leu Glu Tyr Val Leu Asn Tyr Val Lys Lys Gly Val Glu Leu Gly Gly Phe Pro Glu Asp Phe Tyr Lys Ile Leu Ser Arg Pro** |
| original |  | AGG CGT GTT TTG ATT GTT AAT ATT CCT GTG AGG TTG GAT GGT GGT GGT TTT GAG GTG TTT GAG GGT TAT CGT GTG CAG CAT TGT GAT GTT CTC GGC CCG TAT |
| optimized |  | CGG CGG GTG CTC ATC GTG AAC ATC CCC GTG CGG CTC GAC GGG GGG GGG TTC GAG GTG TTC GAG GGG TAC CGG GTG CAG CAC TGC GAC GTG CTC GGG CCC TAC |
| amino acids | 035 | **Arg Arg Val Leu Ile Val Asn Ile Pro Val Arg Leu Asp Gly Gly Gly Phe Glu Val Phe Glu Gly Tyr Arg Val Gln His Cys Asp Val Leu Gly Pro Tyr** |
| original |  | AAG GGT GGG GTT CGT TTT CAT CCT GAG GTT ACT CTT GCC GAC GAC GTC GCC TTG GCT ATT TTA ATG ACG TTG AAA AAT AGC CTA GCT GGC TTG CCG TAT GGC |
| optimized |  | AAG GGG GGG GTG CGG TTC CAC CCC GAG GTG ACC CTC GCC GAC GAC GTG GCC CTC GCC ATC CTC ATG ACC CTC AAG AAC TCC CTC GCC GGG CTC CCC TAC GGG |
| amino acids | 069 | **Lys Gly Gly Val Arg Phe His Pro Glu Val Thr Leu Ala Asp Asp Val Ala Leu Ala Ile Leu Met Thr Leu Lys Asn Ser Leu Ala Gly Leu Pro Tyr Gly** |
| original |  | GGC GCT AAA GGC GCA GTA CGT GTA GAC CCT AAG AAA CTC TCG CAG AGA GAA CTT GAA GAG CTC TCT AGA GGA TAT GCC AGA GCC ATA GCC CCC CTC ATA GGC |
| optimized |  | GGG GCC AAG GGG GCC GTG CGG GTG GAC CCC AAG AAG CTC TCC CAG CGG GAG CTC GAG GAG CTC TCC CGG GGG TAC GCC CGG GCC ATC GCC CCC CTC ATC GGG |
| amino acids | 103 | **Gly Ala Lys Gly Ala Val Arg Val Asp Pro Lys Lys Leu Ser Gln Arg Glu Leu Glu Glu Leu Ser Arg Gly Tyr Ala Arg Ala Ile Ala Pro Leu Ile Gly** |
| original |  | GAC GTA GTC GAC ATC CCA GCG CCT GAC GTT GGG ACA AAC GCC CAA ATC ATG GCG TGG ATG GTA GAC GAA TAT TCA AAA ATA AAG GGC TAC AAC GTG CCC GGG |
| optimized |  | GAC GTG GTG GAC ATC CCC GCC CCC GAC GTG GGG ACC AAC GCC CAG ATC AT GGC CTG GAT GGT GGA CGA GTA CTC CAA GAT CAA GGG GTA CAA CGT GCC CGG |
| amino acids | 137 | **Asp Val Val Asp Ile Pro Ala Pro Asp Val Gly Thr Asn Ala Gln Ile Met Ala Trp Met Val Asp Glu Tyr Ser Lys Ile Lys Gly Tyr Asn Val Pro Gly** |
| original |  | GTA TTC ACC TCA AAA CCA CCA GAG CTA TGG GGA AAC CCA GTG AGA GAA TAC GCC ACA GGC TTT GGA GTA GCA GTA GCG ACA AGA GAA ATG GCA AAA AAA CTA |
| optimized |  | GGT GTT CAC CTC CAA GCC CCC CGA GCT CTG GGG GAA CCC CGT GCG GGA GTA CGC CAC CGG GTT CGG GGT GGC CGT GGC CAC CCG GGA GAT GGC CAA GAA GCT |
| amino acids | 171 | **Val Phe Thr Ser Lys Pro Pro Glu Leu Trp Gly Asn Pro Val Arg Glu Tyr Ala Thr Gly Phe Gly Val Ala Val Ala Thr Arg Glu Met Ala Lys Lys Leu** |
| original |  | TGG GGC GGA ATA GAA GGA AAA ACA GTA GCG ATA CAG GGT ATG GGT AAT GTG GGG AGG TGG ACA GCG TAT TGG CTA GAA AAA ATG GGC GCT AAG GTG ATA GCT |
| optimized |  | CTG GGG GGG GAT CGA GGG GAA GAC CGT GGC CAT CCA GGG GAT GGG GAA CGT GGG GCG GTG GAC CGC CTA CTG GCT CGA GAA GAT GGG GGC CAA GGT GAT CGC |
| amino acids | 205 | **Trp Gly Gly Ile Glu Gly Lys Thr Val Ala Ile Gln Gly Met Gly Asn Val Gly Arg Trp Thr Ala Tyr Trp Leu Glu Lys Met Gly Ala Lys Val Ile Ala** |
| original |  | GTG TCT GAT ATA AAT GGC GTA GCT TAT AGA AAG GAG GGA CTT AAT GTT GAA TTG ATA CAA AAA AAC AAG GGA CTT ACA GGG CCT GCT CTC GTA GAA CTG TTT |
| optimized |  | CGT GTC CGA CAT CAA CGG GGT GGC CTA CCG GAA GGA GGG GCT CAA CGT GGA GCT CAT CCA GAA GAA CAA GGG GCT CAC CGG GCC CGC CCT CGT GGA GCT CTT |
| amino acids | 239 | **Val Ser Asp Ile Asn Gly Val Ala Tyr Arg Lys Glu Gly Leu Asn Val Glu Leu Ile Gln Lys Asn Lys Gly Leu Thr Gly Pro Ala Leu Val Glu Leu Phe** |
| original |  | ACG ACA AAA GAC AAT GCC GAA TTT GTT AAA AAC CCA GAT GCC ATA TTT AAA CTA GAC GTC GAC ATT TTC GTC CCC GCC GCT ATT GAG AAT GTC ATT AGG GGT |
| optimized |  | CAC CAC CAA GGA CAA CGC CGA GTT CGT GAA GAA CCC CGA CGC CAT CTT CAA GCT CGA CGT GGA CAT CTT CGT GCC CGC CGC CAT CGA GAA CGT GAT CCG GGG |
| amino acids | 273 | **Thr Thr Lys Asp Asn Ala Glu Phe Val Lys Asn Pro Asp Ala Ile Phe Lys Leu Asp Val Asp Ile Phe Val Pro Ala Ala Ile Glu Asn Val Ile Arg Gly** |
| original |  | GAT AAT GCT GGG CTT GTG AAG GCT AGG CTT GTT GTT GAG GGT GCT AAT GGC CCT ACT ACT CCT GAG GCT GAG AGG ATT TTG TAT GAG AGG GGG GTT GTC GTA |
| optimized |  | GGA CAA CGC CGG GCT CGT GAA GGC CCG GCT CGT GGT GGA GGG GGC CAA CGG GCC CAC CAC CCC CGA GGC CGA GCG GAT CCTC TAC GAG CGG GGG GTG GTG GTG |
| amino acids | 307 | **Asp Asn Ala Gly Leu Val Lys Ala Arg Leu Val Val Glu Gly Ala Asn Gly Pro Thr Thr Pro Glu Ala Glu Arg Ile Leu Tyr Glu Arg Gly Val Val Val** |
| original |  | GTT CCT GAT ATC CTC GCC AAC GCC GGC GGC GTG ATT ATG TCG TAT TTA GAG TGG GTG GAG AAT CTC CAG TGG TAT ATC TGG GAC GAG GAG GAG ACT AGA AAG |
| optimized |  | GTG CCC GAC ATC CTC GCC AAC GCC GGG GGG GTG ATC ATG TCC TAC CTC GAG TGG GTG GAG AAC CTC CAG TGG TAC ATC TGG GAC GAG GAG GAG ACC CGG AAG |
| amino acids | 341 | **Val Pro Asp Ile Leu Ala Asn Ala Gly Gly Val Ile Met Ser Tyr Leu Glu Trp Val Glu Asn Leu Gln Trp Tyr Ile Trp Asp Glu Glu Glu Thr Arg Lys** |
| original |  | AGA CTT GAA AAC ATA ATG GTA AAC AAC GTC GAG AGA GTA TAC AAG AGA TGG CAG AGA GAA AAA GGA TGG ACA ATG AGA GAC GCA GCA ATA GTA ACC GCA TTA |
| optimized |  | CGG CTC GAG AAC ATC ATG GTG AAC AAC GTG GAG CGG GTG TAC AAG CGG TGG CAG CGG GAG AAG GGG TGG ACC ATG CGG GAC GCC GCC ATC GTG ACC GCC CTC |
| amino acids | 375 | **Arg Leu Glu Asn Ile Met Val Asn Asn Val Glu Arg Val Tyr Lys Arg Trp Gln Arg Glu Lys Gly Trp Thr Met Arg Asp Ala Ala Ile Val Thr Ala Leu** |
| original |  | GAA AGA ATA TAC AAC GCC ATG AAA ATA AGA GGG TGG ATC TAA |
| optimized |  | GAG CGG ATC TAC AAC GCC ATG AAG ATC CGG GGG TGG ATC TGA |
| amino acids | 409 | **Glu Arg Ile Tyr Asn Ala Met Lys Ile Arg Gly Trp Ile ---** |
